# The effects of grafting on the stems of *Eleutherococcus sessiliflorus* were jointly analyzed by transcriptomics and metabolomics

**DOI:** 10.3389/fpls.2026.1824273

**Published:** 2026-07-22

**Authors:** Ming Liang, Ruiqi Wang, Xiaoyan Zhu, Wanying Li, He Yang, Mingjie Song, Xiujuan Lei, Yingping Wang, Nanqi Zhang

**Affiliations:** 1 College of Traditional Chinese Medicine, Jilin Agricultural University, Changchun, China; 2 State Local Joint Engineering Research Center of Ginseng Breeding and Application, Changchun, China

**Keywords:** amino acid, *Eleutherococcus sessiliflorus*, grafting, multi-omics technologies, unsaturated fatty acids

## Abstract

**Background:**

This study investigates the core molecular mechanisms by which the rootstock of *Eleutherococcus sessiliflorus* (*E.sessiliflorus*) regulates the stem growth of *Eleutherococcus senticosus* (*E.senticosus*), using *E.senticosus* as the scion.

**Methods:**

Grafted plants with *E.sessiliflorus* as the rootstock served as the primary research subjects, while self-rooted *E.senticosus* plants were utilized as controls. An integrated approach, combining physiological and biochemical assays, transcriptomics, and metabolomics, was employed to analyze the mechanisms, with verification by qRT-PCR.

**Results:**

Physiological and biochemical analyses showed that self-grafting had no significant effect on *E. senticosus* stems. By contrast, cross-root grafting did not significantly alter moisture content, total ash, or extractive content. However, it significantly enhanced nutrient accumulation and antioxidant defense capacity in the stems, thereby promoting secondary metabolite accumulation. Transcriptomic and metabolomic analyses further revealed that grafting significantly regulates the biosynthesis pathways of unsaturated fatty acids and amino acids. Differential expression of key enzymes, such as SCD, FAD2, icd, and PK, facilitates metabolic specialization and collaborative adaptation between rootstock and scion.

**Conclusions:**

This study elucidates potential core targets and molecular mechanisms underlying the influence of grafting on *E.senticosus*, providing valuable theoretical references and research directions for variety improvement, high-quality cultivation, and the sustainable utilization of rare medicinal plant resources of *E.senticosus*.

## 1 Introduction

Wild medicinal plants have been depleted due to excessive harvesting, while artificial cultivation faces significant challenges, including varietal degradation, low concentrations of active compounds, and poor stress resistance. These issues hinder the ability to satisfy the market’s demand for high-quality medicinal materials. Grafting is a mature technique in traditional agriculture for improving plant traits. By taking advantage of the complementary traits of rootstock and scion, it promotes growth and development (Han et al., 2024), improves fruit yield and quality (Wang et al., 2025), enhances abiotic stress tolerance (Guo et al., 2023), resists viral attacks (Dong et al., 2025), increases energy utilization (Liang et al., 2021), modifies soil microbial composition (Yavuz et al., 2025), and boosts economic value. Therefore, it is widely used in fruits, vegetables, forests, and ornamental flowers. Grafting is widely used in traditional agriculture. However, its application in medicinal plant cultivation remains relatively limited. Introducing this technique into medicinal plant cultivation and exploring its regulatory effects on the accumulation of active ingredients and growth characteristics of medicinal materials (Ma, 2007; Kul and Öztürk, 2024), have important theoretical value and practical significance. This can help address resource challenges in medicinal plants and promote the high-quality development of the traditional Chinese medicine industry. The cross-border application of this technology in medicinal plant cultivation, coupled with the exploration of its regulatory effects on the accumulation of bioactive constituents and growth characteristics of medicinal materials, holds significant theoretical value and practical significance for addressing the resource predicament of medicinal plants and promoting the high-quality development of the traditional Chinese medicine industry.

*Eleutherococcus senticosus* (Rupr. & Maxim.) Maxim. (E. senticosus) is a traditional Chinese medicine belonging to the Araliaceae family and the genus Eleutherococcus. This plant is both edible and medicinal, and it contains a diverse array of chemical constituents. It is known for its effects in tonifying qi, strengthening the spleen, nourishing the kidney, and calming the mind. Currently, the *E.senticosus* industry faces two major challenges. First, wild resources are becoming depleted. Prolonged and unregulated harvesting has led to wild *E.senticosus* being listed as a second-class protected plant in China. Second, artificial cultivation and propagation encounter problems such as slow growth, varietal degradation, and inconsistent levels of active ingredients. Moreover, these plants have strict environmental requirements and exhibit relatively low resistance to cold, drought, and various pests and diseases (Wang et al., 2023). Our research group previously selected *Eleutherococcus sessiliflorus* (Rupr. & Maxim.) S.Y. Hu (E. sessiliflorus) as the rootstock for grafting. This species belongs to the same family and genus as *E.senticosus* and was chosen due to its vigorous growth, well-developed root system, and strong stress resistance. The genetic compatibility between these closely related species results in a grafting survival rate of 70%. After grafting, the plants show improved growth performance and yield, characterized by enhanced nutrient reserves and stress resistance. Additionally, the leaves exhibit increased levels of primary metabolites (e.g., sugars and amino acids), elevated triterpenoid content, and upregulated expression of genes involved in chlorogenic acid and triterpenoid biosynthesis (Wang et al., 2023). Since leaves are photosynthetic organs, they provide only an initial indication of the effects of grafting on nutrient synthesis. In contrast, the stem plays a crucial role in nutrient storage and transformation (Li et al., 2023; Zhao et al., 2025). The stem is also the primary medicinal part of *E.senticosus.* Investigating the grafted *E.senticosus* stem helps to elucidate the complete physiological chain: nutrients absorbed by the rootstock are assimilated through leaf photosynthesis, and the resulting products ultimately accumulate and are transformed in the stem. This research aims to clarify the regulatory pathways governing material exchange and signal transduction between rootstock and scion, particularly regarding stem growth and the synthesis of active ingredients. Thus, this study focused on grafted plants of *E.senticosus* (scion)×*E.sessiliflorus* (rootstock) as the subjects of investigation. Self-propagated plants of *E.senticosus* and *E.sessiliflorus*, along with seedlings of the original species, were utilized as controls to comprehensively assess the impact of grafting on scions through an analysis of physiological and biochemical markers. Based on this foundation, we employed a combined analysis strategy integrating transcriptomics and non-targeted metabolomics. Screen grafting-responsive signature metabolites, identify the core metabolic pathways regulating stem morphological and physiological changes in *E.senticosus*, and mine differentially expressed genes involved in the synthesis, transport, and regulation of target metabolites. The results of this study can clarify the core molecular mechanisms underlying the regulation of stem growth in *E.senticosus* by *E.sessiliflorus* rootstocks, and identify key candidate targets for rootstock-scion interactions. This achievement provides a molecular model for research on rootstock-scion interactions in Araliaceae and perennial woody medicinal plants, filling the gap in the theoretical understanding of molecular regulation of grafting in medicinal plants. In subsequent studies, techniques such as gene editing and marker-assisted selection can be employed to directionally improve *E.senticosus* cultivars, addressing the challenges associated with traditional breeding. Meanwhile, these findings can be extended to closely related species within the same family, providing technical support for the resource restoration and alternative cultivation of rare and endangered medicinal plants, and alleviating the harvesting pressure on wild populations.

## 2 Materials and methods

### 2.1 Plant materials

The plant grafting experiment was conducted in May 2023 at the Facility Agriculture Base of Jilin Agricultural University (43°48′N, 125°24′E). Using the same cleft grafting method at the same time, *E.sessiliflorus* was used as the scion to perform autografting (*E.sessiliflorus*/*E.sessiliflorus*) and heterografting (*E.sessiliflorus*/*E.senticosus*) (**Supplementary Figure 1**). Samples were collected in September of the following year (the optimal harvest period for *E.sessiliflorus* stems). A total of five sample groups were collected: heterograft scions of *E.senticosus* (ES-HS), heterograft rootstocks of *E.sessiliflorus* (ESs-HR), autograft scions of *E.senticosus* (ES-AS), untreated seedling stem segments of *E.senticosus* (ES-SS), and untreated seedling stem segments of *E.sessiliflorus* (ESs-SS). The last two groups, which were matched for growth vigor and height with the grafted plants, served as controls. All samples were flash-frozen in liquid nitrogen and stored at -80 °C for subsequent use.

### 2.2 Evaluation of physiological and biochemical indicators

Quality control indicators, including Moisture content (MC), Extractive content (EC), and Total ash content (TAC), were assessed following the methodologies outlined in the *Pharmacopoeia of the People’s Republic of China* (hereafter referred to as “ *Chinese Pharmacopoeia*”) (Commission, 2025). The determination of Syringin content (SYC) was performed using the Thermo Fisher Scientific UHPLC-Q-Exactive Orbitrap mass spectrometer. For chromatographic separation, the Accucore RP-MS column (100×2.1 mm, 2.6 μm) was employed. The mobile phase consisted of acetonitrile (phase A) and a 0.1% formic acid aqueous solution (phase B). The gradient elution program was established as follows: maintain 80% of phase B from 0 to 8 minutes. A standard curve was generated under the aforementioned chromatography-mass spectrometry conditions, with linear regression analysis conducted using the concentration of the syringin standard as the abscissa and the corresponding peak area as the ordinate. Sample extraction was performed according to the methods outlined in the *Chinese Pharmacopoeia*. The extract was subsequently filtered using a 0.22 μm organic phase filter membrane, and the SYC content was quantified under instrument conditions consistent with those used for standard curve generation.

Antioxidant indicators, the Malondialdehyde (MDA) content was determined using the Thiobarbituric Acid (TBA) method, calculated based on the difference in absorbance at 600 nm and 532 nm. Peroxidase (POD) activity was assayed by the Guaiacol method, measuring the change in absorbance at 470 nm per minute. Superoxide Dismutase (SOD) activity was measured using the WST-8 method by detecting the absorbance at 450 nm. The assays for MDA, POD, and SOD were performed according to the instructions provided in the kits from Suzhou Geris Biotechnology Co., Ltd. Catalase (CAT) activity was determined following the kit instructions from Beijing Solarbio Science & Technology Co., Ltd., using the UV absorption method at 240 nm.

Nutrient indicators, the Soluble Sugar (SS) content was determined using the anthrone-sulfuric acid method by measuring the absorbance at 620 nm. The Starch content (SC) was determined via the acid hydrolysis method, also by measuring the absorbance at 620 nm. The Soluble Protein (SP) content was determined using the Coomassie Brilliant Blue G-250 method by measuring the absorbance at 600 nm. For all the above indicators, the specific procedures for sample extraction and experimental operations were carried out in accordance with the instructions of the respective kits supplied by Suzhou Geris Biotechnology Co., Ltd.

Secondary metabolite indicators were assessed using 60% ethanol as the extraction solvent. The extraction solution was prepared at a ratio of 1:20 between the material and the solvent, followed by ultrasonic extraction at 50°C for 35 minutes (100W). After centrifugation, the supernatant was collected (4°C, 8000r/min, 10min). This extraction process was repeated twice, and the two supernatants were combined for subsequent analysis. Hyperoside, Gallic acid, Syringin, and Leucine served as standard substances. Color development was performed using the Aluminum salt method (Liu et al., 2021), Folin-Ciocalteu method (Graczyk et al., 2022), Vanillin-concentrated sulfuric acid method (Chen et al., 2022), and Ninhydrin method (Jabeen et al., 2015). Absorbance was measured at wavelengths of 510 nm, 760 nm, 544 nm, and 570 nm. The concentration of each substance in the samples was determined using standard curves.

### 2.3 Transcriptomics measurement and analysis

Sample pretreatment: Total RNA extraction was performed following the manufacturer’s instructions (Invitrogen), employing TRIzol^®^ reagent for RNA isolation from tissues, while DNase I (TaKara) was utilized to eliminate genomic DNA. The quality of the RNA was assessed using the 2100 bioanalyzer (Agilent) and quantified with the ND-2000 (NanoDrop Technologies). High-quality RNA samples were selected based on the following criteria: OD260/280 = 1.8 ~ 2.2, OD260/230 ≥ 2.0, RIN ≥ 6.5, 28S:18S ≥ 1.0, and a minimum yield of 10μg. These samples were then used to construct sequencing libraries. Following the TruSeqTM RNA Sample Preparation Kit protocol from Illumina (San Diego, California), the RNA-Seq transcriptome library was prepared using 1μg of total RNA. The original paired-end reads were trimmed with Trimmomatic software, and the clean data underwent *de novo* RNA assembly using Trinity software. Functional annotations were derived by comparing the assembled transcripts against databases such as NCBI Protein Non-redundancy. Gene Ontology (GO) annotations were obtained through the BLAST2GO program, and metabolic pathway analysis was performed using Kyoto Encyclopedia of Genes and Genomes (KEGG).

Differential expression analysis and functional study: Transcript expression levels were calculated using the RPKM method, while gene and isoform abundance was quantified with RSEM software. Differentially expressed genes (DEGs) were screened using thresholds of |log_2_^Fold change^| ≥ 1 and P < 0.05. GO functional enrichment and KEGG pathway analyses were performed using Goatools and KOBAS, respectively.

WGCNA construction: WGCNA constructs the network using the absolute values of the Pearson correlation coefficient. Through a series of steps, it generates the adjacency matrix, calculates the topological overlap distance, and performs clustering. The cutreeDynamic function is employed to define the minimum module containing 30 genes. Modules with high similarity are merged using a threshold of 0.25, while meaningful edges are identified with a weight value of 0.10. The analysis focuses on the correlation between the characteristic genes of the module and traits. Genes within the top 15% of connection numbers are designated as hub genes.

qRT-PCR verification was conducted using cDNA synthesized through reverse transcription of the same RNA utilized for RNA-Seq, following the protocol of the Sisco Bioscience Kit. Fluorescence quantification was performed on a selection of relevant genes. The transcriptional levels of the target genes were quantitatively assessed by comparing the cycle threshold (Ct) values required for different samples to reach the fluorescence threshold, with the *GAPDH* (*Glyceraldehyde 3-phosphate dehydrogenase*) gene serving as the internal reference (Liu et al., 2020; Ye et al., 2021; Wang et al., 2023). The relative expression levels of the genes were calculated using the 2^-ΔΔCt^ method (**Supplementary Table 1** provides all pertinent information regarding the qRT-PCR primers).

### 2.4 Metabolomics measurement and analysis

Sample pretreatment: A total of 100 mg of each sample was ground using liquid nitrogen, homogenized, and resuspended in pre-cooled 80% methanol containing 0.1% formic acid. The resulting mixture was vortexed thoroughly at a ratio of 1:4. Samples were then incubated on ice for 5 minutes before being centrifuged at 4 °C and 15,000 revolutions per minute for an additional 5 minutes. A portion of the supernatant was taken and diluted with LC-MS grade water to achieve a final methanol concentration of 53%. The sample was subsequently transferred to a new centrifuge tube and centrifuged again at 4 °C and 15,000 revolutions per minute for 10 minutes. Finally, the supernatant was collected and filtered through a membrane for future use (Yuan et al., 2012; Barri and Dragsted, 2013).

Analysis conditions for UHPLC-MS/MS: The Vanquish ultra-high performance liquid chromatography system (Thermo Fisher Scientific, Germany) was utilized in conjunction with the Orbitrap Q ExactiveTM HF mass spectrometer (Thermo Fisher Scientific, Germany) for the analysis. The chromatographic column employed was Hypesil Gold (100×2.1 mm, 1.9μm). The flow rate was set at 0.2 ml/min. For the mobile phase, in positive ion mode, a solution of 0.1% formic acid in water (A) was used in combination with methanol (B); in negative ion mode, 5 mM ammonium acetate at pH 9.0 (A) was paired with methanol (B). The gradient was as follows: from 0 to 1.5 minutes, 2% B; from 1.5 to 12 minutes, 2-100% B; from 12 to 14 minutes, 100% B; from 14 to 14.1 minutes, 100-2% B; and from 14.1 to 17 minutes, 2% B. The electrospray ion source was employed for scanning both positive and negative ions, with a spray voltage of 3.2 kV, a capillary temperature of 320°C, a sheath gas flow rate of 40 arb, and an auxiliary gas flow rate of 10 arb.

Metabolomics data were preprocessed using Compound Discoverer 3.1 software, including baseline filtering, peak identification, integration, retention time correction, peak alignment, and normalization. Metabolite identification was performed by matching detected retention times, parent/daughter ions, and secondary fragmentation information against HMDB, Lipidmaps, METLIN, and PMDB databases. Using Python and CentOS systems, further matching with mzCloud, mzVault, and MassList databases yielded accurate qualitative and relative quantitative results. Statistical analysis was performed using SIMCA software (version 14.1), employing multivariate analysis through PCA and OPLS-DA. Metabolite P-values were calculated via univariate t-tests, with differential metabolites selected based on the threshold of VIP > 1, P < 0.05, and fold change ≥ 2 or ≤ 1/2. For result visualization, log_2_^FoldChange^ and -log_10_^p-value^ analyses were used to assess metabolic trend changes. Correlations among differentially expressed metabolites were analyzed using Pearson’s correlation coefficient (cor() function), with P < 0.05 considered statistically significant. Annotation and pathway analysis relied on KEGG, HMDB, and LIPID Maps databases. Metabolic pathway enrichment analysis was performed via KEGG; pathways were deemed significantly enriched when the proportion of differentially expressed metabolites exceeded the background level and P < 0.05.

### 2.5 Statistical analysis

In this study, physiological and biochemical indices were measured with three biological replicates and three technical replicates. Significance analysis was performed using SPSS 25.0 statistical software, and different lowercase letters (a, b, c) indicate significant differences between groups (P < 0.05). Visualization analysis was conducted using Origin 2021. For transcriptomics and metabolomics, six biological replicates were used. In transcriptomics, qRT-PCR validation was performed with three biological replicates and three technical replicates. For metabolomics, quality control (QC) samples were interspersed to monitor instrument stability, and multivariate statistical analysis was carried out using SIMCA 14.1.

## 3 Results and analysis

### 3.1 Evaluation results of physiological and biochemical indicators

**Figure 1:**
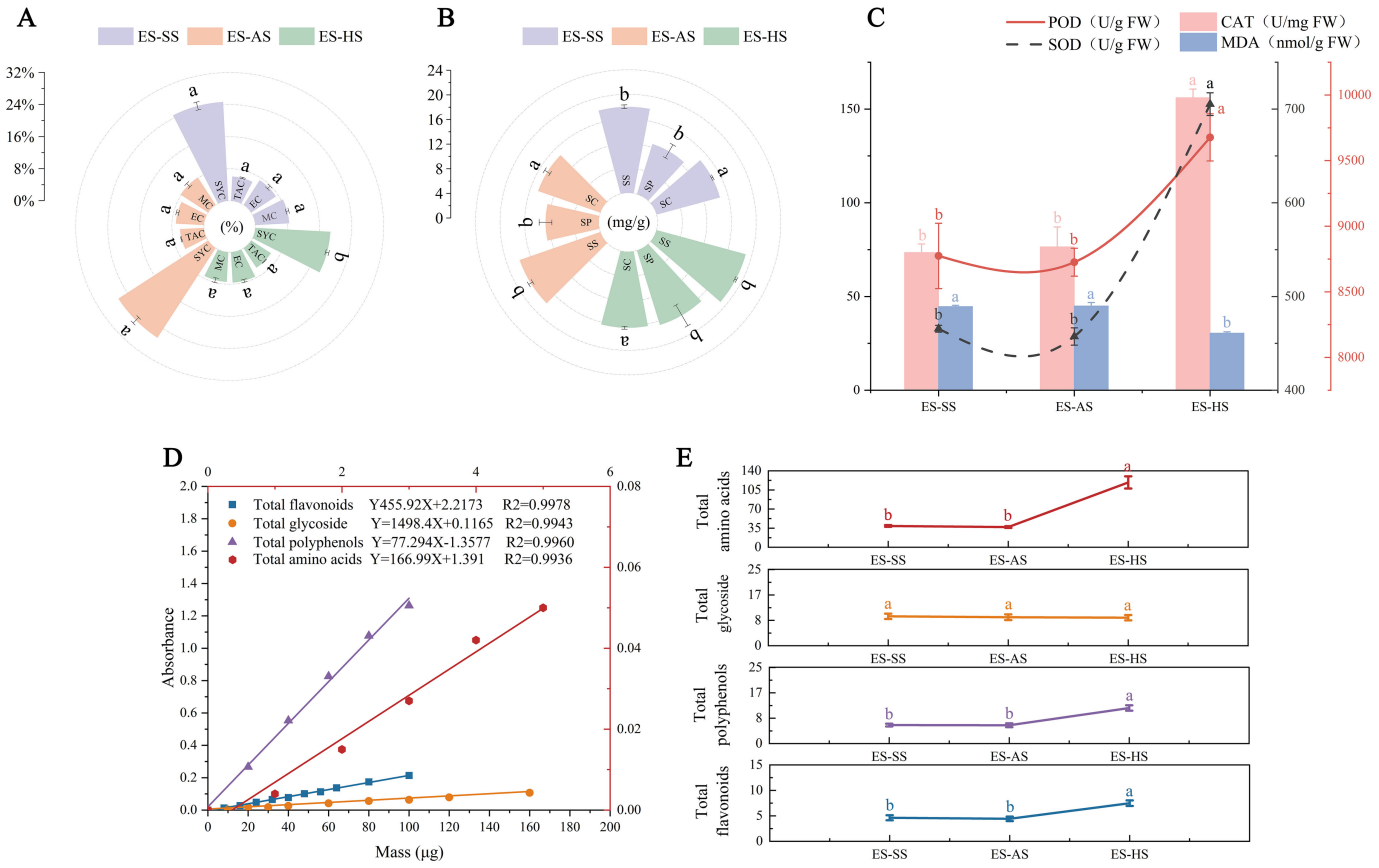
Grafting effects on E.senticosus stems. Physicochemical indices **(A)**; Nutrients **(B)**; Antioxidant activity **(C)**; Standard curve **(D)**; Secondary metabolites **(E)**.

The results showed that in the ES-SS comparison group, the grafting treatment groups (ES-AS and ES-HS) exhibited no statistically significant differences in MC, EC, and TAC (P > 0.05). Specifically, the maximum MC was 8.59%, the minimum EC was 7.12%, and the maximum TAC was 6.17%. All index values adhered to the relevant standards outlined in the *Chinese Pharmacopoeia* (MC ≤ 10%, EC ≥ 3%, TAC ≤ 9%). The linear regression equation for the standard curve of syringin is given by Y = 4.31 × 10^6^X + 1.33 × 10^7^. The SYC in the ES-SS, ES-AS, and ES-HS groups were 0.25%, 0.27%, and 0.19%, respectively. Statistical analysis revealed no significant difference in SYC between the ES-SS and ES-AS groups (P > 0.05). However, the SYC in the ES-HS group decreased significantly (P < 0.01) but still met the minimum standard established in the *Chinese Pharmacopoeia* (≥ 0.05%) (**Figure 1A**).

At the nutrient level, no significant differences were observed in the contents of SC, SP, and SS between ES-SS and ES-AS (*P* > 0.05). In contrast, the aforementioned indices in ES-HS exhibited significant changes. Specifically, the SC content was 1.16 times higher than those in ES-SS and ES-AS, respectively; the SP content was 1.49 and 1.47 times higher than those in the two groups, respectively; and the SS content also increased significantly, reaching 1.08 and 1.07 times those of the two groups, respectively (**Figure 1B**).

In the assessment of antioxidation-related indicators, the ES-HS exhibited a marked advantage in antioxidant enzyme activity, demonstrating a significantly superior overall antioxidant defense capability compared to the other two groups. Specifically, the activities of CAT, POD, and SOD in the ES-HS were 2.13 times, 1.51 times, and 1.10 times greater than those in the ES-SS, respectively, and 2.05 times, 1.54 times, and 1.11 times greater than those in the ES-AS. Concurrently, the MDA content in the ES-HS group decreased to 0.68 times that of both the ES-SS and ES-AS (**Figure 1C**).

The standard curves of hyperoside, gallic acid, ginsenoside Re and leucine are shown in **Figure 1D**. All standard curves exhibited excellent linear relationships, with the coefficient of determination (R²) all greater than 0.99, confirming that the quantitative analysis method in this study has good reliability. The content changes of the above indicators in different treatment groups are shown in **Figure 1E**: Compared with ES-AS, there were no significant changes in the four secondary metabolites of ES-SS (p > 0.05). Compared with the control group (ES-SS and ES-AS), except for no significant difference in total saponin content (p > 0.05), the contents of total flavonoids, total polyphenols and total amino acids of ES-HS were significantly increased (P < 0.05).

In conclusion, heterojunction treatment has a significant differentiated regulatory effect on the physiological metabolism of *E.senticosus*. Based on this, samples from the ES-SS, ES-HS, ESs-SS and ESs-HR were subsequently selected for combined transcriptomic and metabolomics analysis.

### 3.2 Transcriptomic analysis results

#### 3.2.1 Overview of transcriptome data

**Figure 2:**
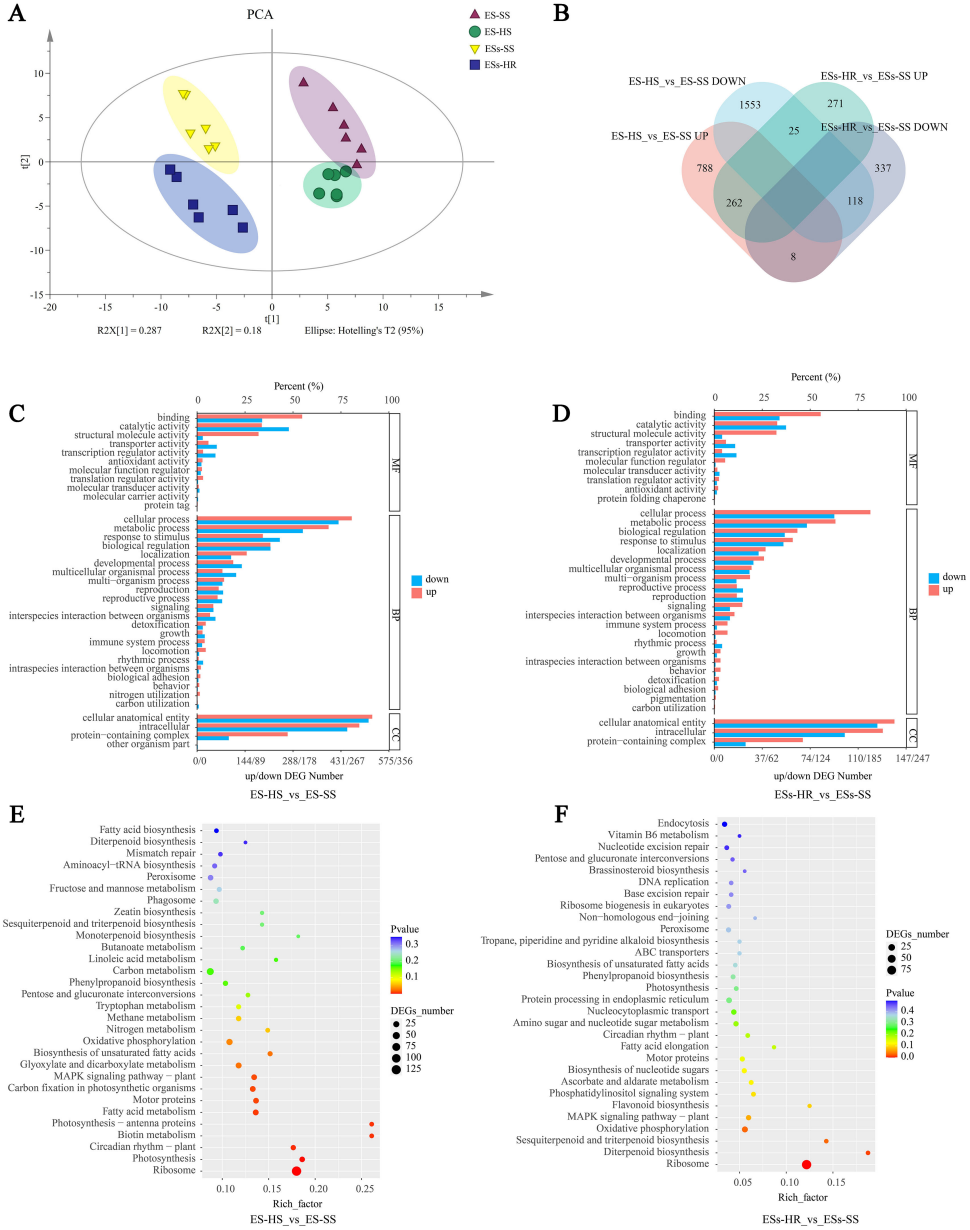
PCA Analysis **(A)**, Venn Diagram of DEGs **(B)**, and GO **(C, D)** KEGG **(E, F)** Enrichment Analysis.

After quality control, the Q30 was ≥ 94.00% and the Mapped Rate was ≥ 60.00%, which met the commonly accepted qualification criteria for RNA-seq data processing and analysis (Consortium, 2014; Conesa et al., 2016), indicating that the data quality was suitable for downstream analyses (**Supplementary Tables 2**, **S3**). The PCA plot based on transcriptome data (**Figure 2A**) illustrates that the four sample groups are distinctly separated, while the six biological replicates within each group cluster together. This observation indicates significant differences among the samples and demonstrates good sample reproducibility. From the PC1 axis, ES-SS and ES-HS were distributed in the positive half axis of PC1, while ESs-SS and ESs-HR were distributed in the negative half axis of PC1, indicating that there were significant differences in the overall transcription level between the two control groups. From the PC2 axis, ES-SS and ES-HS also showed obvious upper and lower separation, indicating that the rootstock had a certain effect on the scion. The screening threshold was set at |log_2_^FoldChange^ | ≥1 and p < 0.05 to identify DEGs. That the number of DEGs in the two groups was 2754 (with 1058 up-regulated and 1696 down-regulated) and 1021 (with 558 up-regulated and 463 down-regulated), respectively. Notably, there were 413 overlapping genes between ES-HS_*vs*_ES-SS and ESs-HR_*vs*_ESs-SS. Among these, 262 genes exhibited an up-regulation trend, 118 genes displayed a down-regulation trend, and 33 genes showed opposing up-regulation trends in the two control groups (**Figure 2B**).

#### 3.2.2 Enrichment analysis of DEGs

The biological functions of these DEGs were further annotated through GO and KEGG enrichment analyses. Enrichment analysis was performed using the software Goatools (https://github.com/tanghaibao/GOatools) using Fisher ‘s exact test. In order to control the calculated false positive rate, the FDR multiple test method was used to correct the p value. When p < 0.05, it was considered that this GO function had significant enrichment. GO terms were functionally categorized into three groups: Biological Process (BP), Cellular Component (CC), and Molecular Function (MF). A total of 743 and 599 GO terms were annotated in the two comparison groups, respectively. As shown in **Figures 2C, D**, the functional category terms of the two groups were highly similar: CC was mainly enriched in cellular anatomical entity, intracellular, and protein-containing complex; MF was significantly enriched in binding, catalytic activity, and structural molecule activity in both groups; BP was significantly enriched in cellular process, metabolic process, and biological regulation in both groups. To gain deeper insights into the grafting mechanism, we further performed KEGG enrichment analysis (**Figures 2E, F**). KEGG pathways in each group were screened based on a p-value threshold of p < 0.05, and bubble plots were generated. Consistent with the GO enrichment results, nearly 70% of the enriched pathways were shared between the two groups, such as Photosynthesis, Fatty acid metabolism, MAPK signaling pathway-plant, Biosynthesis of unsaturated fatty acids, and Tryptophan metabolism. These results indicate that heterografting with non-homologous rootstocks may comprehensively participate in energy supply, substance transport, signal transduction, and metabolic phenotype remodeling of *E.senticosus* after grafting by regulating the coordinated operation of the aforementioned core pathways.

#### 3.2.3 Analysis of weighted gene co-expression network of grafting response genes

**Figure 3:**
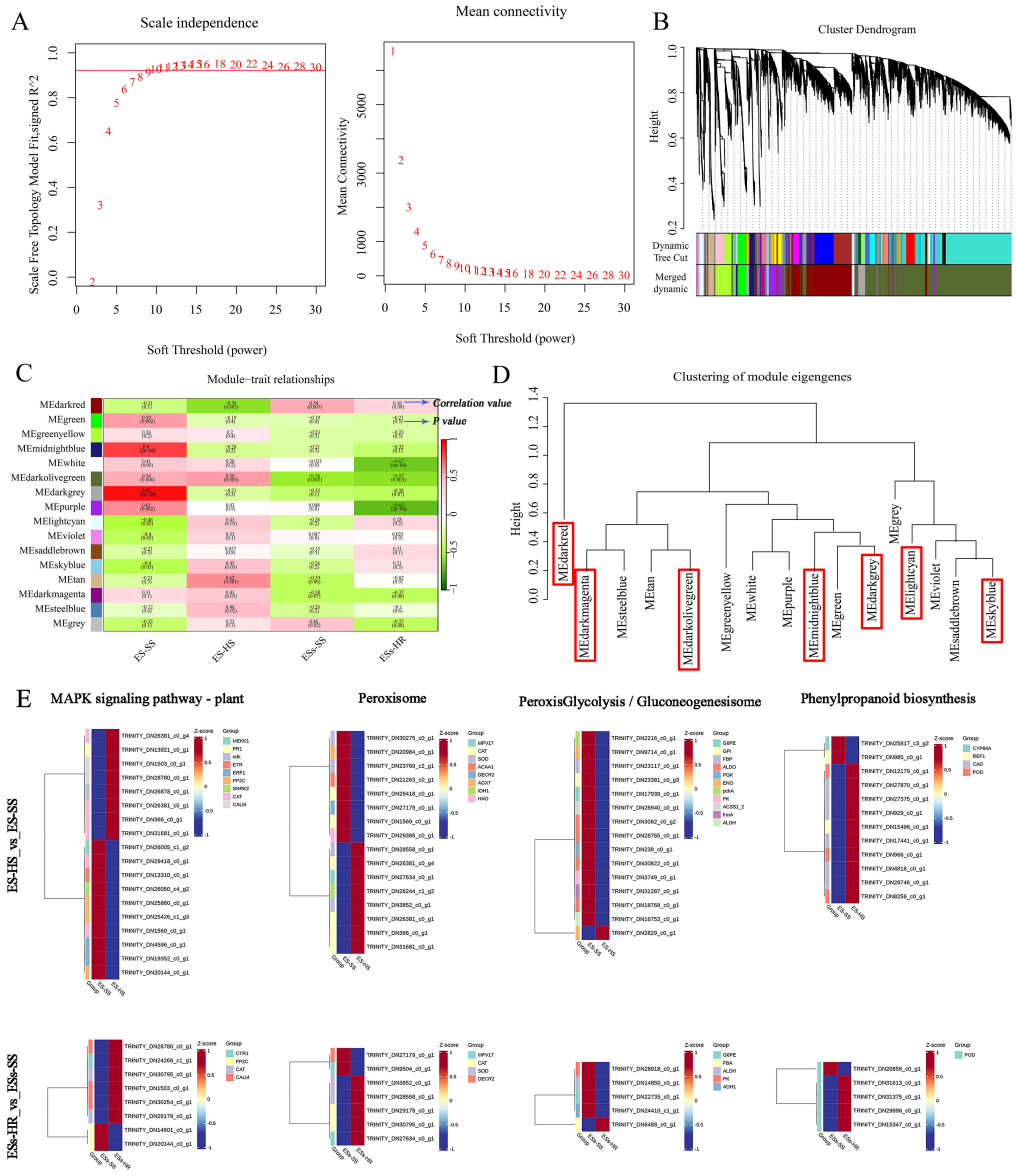
WGCNA of genes under grafting effects.

To identify hub genes involved in key substance transport before and after grafting, weighted gene co-expression network analysis (WGCNA) was performed on all genes. The soft threshold was set to 22 (**Figure 3A**) to construct a scale-free network topology and assess the average gene connectivity within the network. Based on RPKM expression values, a gene co-expression network was constructed, and a total of 16 distinct co-expression modules were obtained from all sample genes (**Figure 3B**). Subsequently, we calculated the correlation coefficients between each module and different samples, and finally screened out seven core modules with important research implications (**Figure 3C**). The correlation coefficient between the calculation module and the sample was calculated, and 7 core modules with research value were selected (**Figure 3D**). Among them, the dark red, dark olive green and dark magenta modules exhibited distinctly opposite expression trends between ES-HS and ESs-HR. The light cyan and sky blue modules showed synergistic positive correlation characteristics, while the midnight blue and dark grey modules presented synergistic negative correlation patterns. Genes were ranked according to their intranetwork connection degrees, and the top 30 genes were defined as hub genes. Functional enrichment analysis was further performed on these seven core modules (**Supplementary Figure 2**), and the results revealed that these modules were commonly enriched in ten metabolic pathways, including “Glycolysis/Gluconeogenesis”, “Biosynthesis of amino acids”, “MAPK signaling pathway-plant”, and “Phenylpropanoid biosynthesis”. By combining the KEGG and WGCNA results, a total of four key pathways were identified, namely MAPK signaling pathway-plant, peroxisome, glycolysis/gluconeogenesis, and phenylpropanoid biosynthesis, involving 71 distinct genes (**Supplementary Table 5**). A hierarchical clustering heatmap was used to visualize the expression levels of genes associated with these target pathways, and the results are shown in **Figure 3E**. To verify the accuracy of the RNA-seq data, 8 DEGs were randomly selected, and their expression patterns were confirmed by qRT-PCR. The results showed that the expression patterns of these genes were highly consistent with the RNA-seq data (**Supplementary Figure 3**), indicating that the transcriptomic data in this study had good reproducibility and reliability. These findings suggest that the 7 identified core modules and 10 enriched pathways may collectively form the molecular regulatory network underlying key substance transport before and after grafting.

### 3.3 Metabolomics analysis results

#### 3.3.1 Quality assessment of metabolomics data

**Figure 4:**
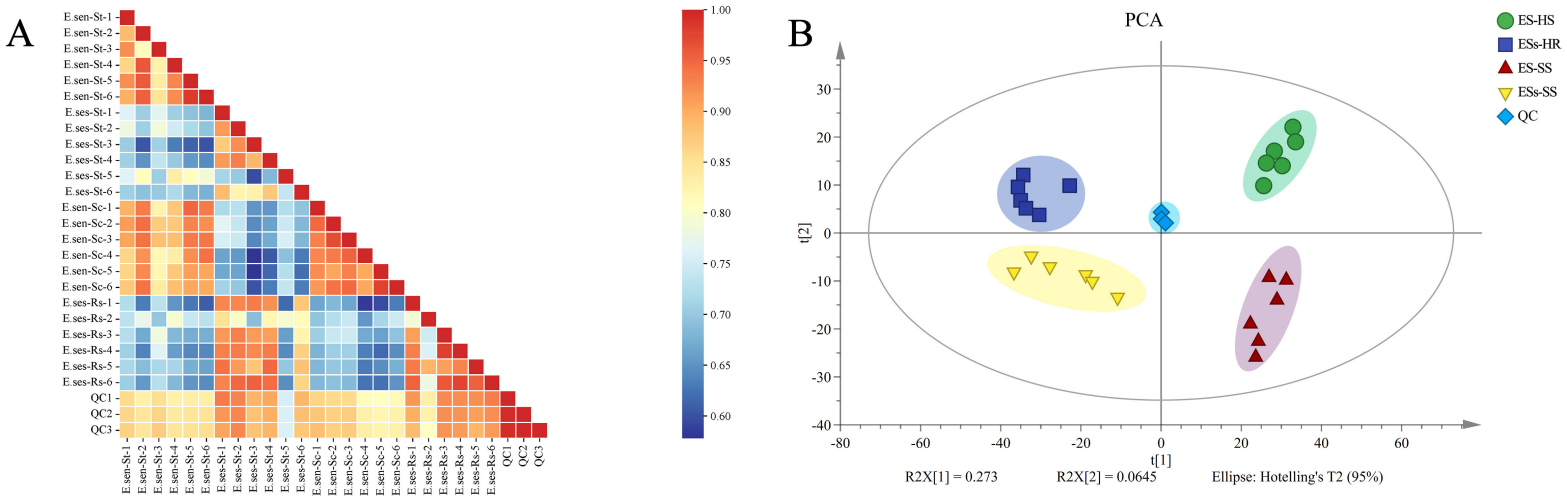
Differential metabolite multivariate statistical analysis. Matrix heatmap **(A)**; PCA **(B)**.

Total Ion Chromatograms (TICs) from metabolomic analysis demonstrated that the analytical instrument employed exhibited high signal intensity, sufficient peak capacity, and excellent reproducibility of metabolite retention times (**Supplementary Figure 4**). This indicated a stable and reliable experimental detection system, laying a solid foundation for subsequent data analysis. By matching information from multiple metabolite databases and integrating characteristics including mass-to-charge ratio (m/z), retention time, and tandem mass spectrometry (MS/MS) fragment ions, a total of 2,794 metabolites were identified across the 4 sample groups (positive: 1777, negative: 1017). Results of matrix heatmap analysis (**Figure 4A**) showed that samples within the same group were predominantly colored red/orange, indicating a high correlation of metabolite expression among intra-group samples and verifying the stability of biological replicates. In contrast, significant differences in correlation were observed between inter-group samples, providing a prerequisite for the subsequent screening of DAMs. The PCA model further reflected the overall distribution characteristics of the data (**Figure 4B**), confirming significant metabolic differences between groups and reliable sample reproducibility. Based on the aforementioned quality control and overall difference analysis results, OPLS-DA models were constructed for the ES-HS_*vs*_ES-SS and ESs-HR_*vs*_ESs-SS (**Supplementary Figure 5**). These models achieved effective separation of samples in each comparison group; cross-validation indicated that the R²Y and Q² values of the models met reliability requirements without overfitting. Thus, the experimental results are stable and credible, and can be used for the subsequent screening and identification of DAMs.

#### 3.3.2 Screening for DAMs

**Figure 5:**
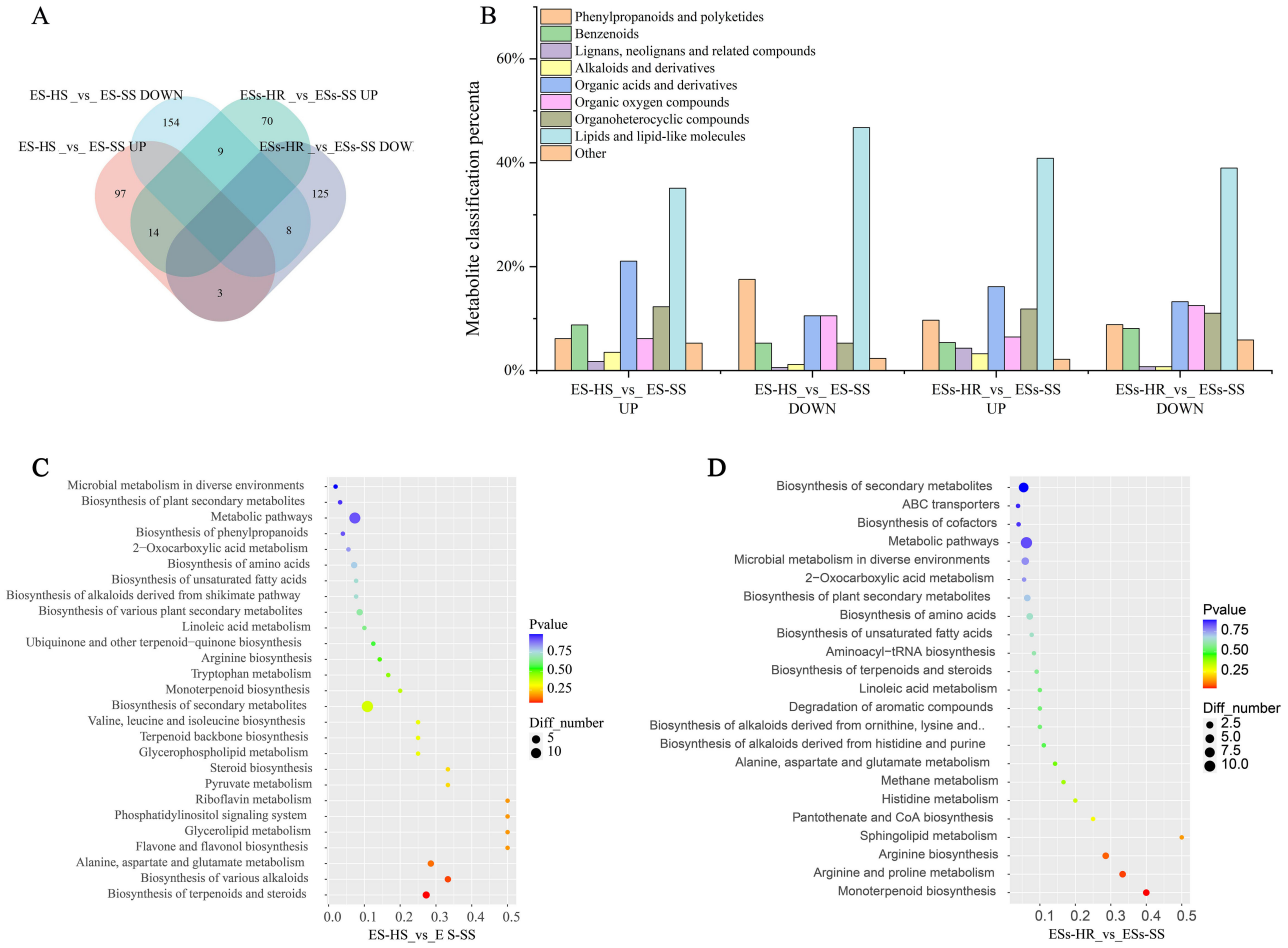
DAMs Screening **(A)**, Metabolite Classification **(B)** and KEGG Pathway Enrichment Analysis **(C, D)**.

DAMs between the two comparison groups were screened from the aforementioned differential metabolites using preset thresholds. A Venn diagram was generated to visualize the DAMs across different comparison groups (**Figure 5A**). ES-HS_*vs*_ES-SS comparison identified 114 upregulated metabolites and 171 downregulated metabolites. The upregulated DAMs were mainly categorized into Lipids and lipid-like molecules (35.09%), Organic acids and derivatives (21.05%), and Organoheterocyclic compounds (12.28%), etc.; while the downregulated DAMs were predominantly classified as Lipids and lipid-like molecules (46.78%), Phenylpropanoids and polyketides (17.54%), and Organic acids and derivatives (10.53%), etc (**Figure 5B**, **Supplementary Table 6**). For the ESs-HR_*vs*_ESs-SS comparison, 93 metabolites were found to be upregulated and 136 downregulated. The upregulated DAMs primarily included Lipids and lipid-like molecules (40.86%), Organic acids and derivatives (16.13%), and Organoheterocyclic compounds (11.83%), etc.; the downregulated DAMs mainly covered Lipids and lipid-like molecules (38.97%), Phenylpropanoids and polyketides (13.24%), and Organic oxygen compounds (12.50%), etc (**Figure 5B**, **Supplementary Table 7**). The results showed that 14 DAMs were significantly up-regulated in both groups, 8 DAMs were significantly down-regulated in both comparison groups, and 12 DAMs exhibited opposite expression trends between the two groups (**Supplementary Table 8**).

#### 3.3.3 KEGG pathway analysis of DAMs

KEGG pathway enrichment analysis was performed separately on the DAMs of the two groups. The results showed that a total of 27 significant pathways were enriched in the ES-HS_*vs*_ES-SS group (**Figure 5C**), while 23 significant pathways were enriched in the ESs-HR_*vs*_ESs-SS group (**Figure 5D**). Further intersection analysis revealed that 12 pathways were significantly enriched in both groups, including Arginine biosynthesis, Alanine, aspartate and glutamate metabolism, Biosynthesis of unsaturated fatty acids, and Monoterpenoid biosynthesis. These findings suggest that the rootstocks used in heterografting may coordinate the operation of the above-mentioned core pathways to jointly participate in substance transport, metabolic reconstruction, and the biosynthesis of pharmacologically active components in *E.senticosus* after grafting. This study provides key pathway-level evidence for elucidating the regulatory mechanism of rootstocks on the metabolic.

### 3.4 Combined analysis of transcriptomics and metabolomics

**Figure 6:**
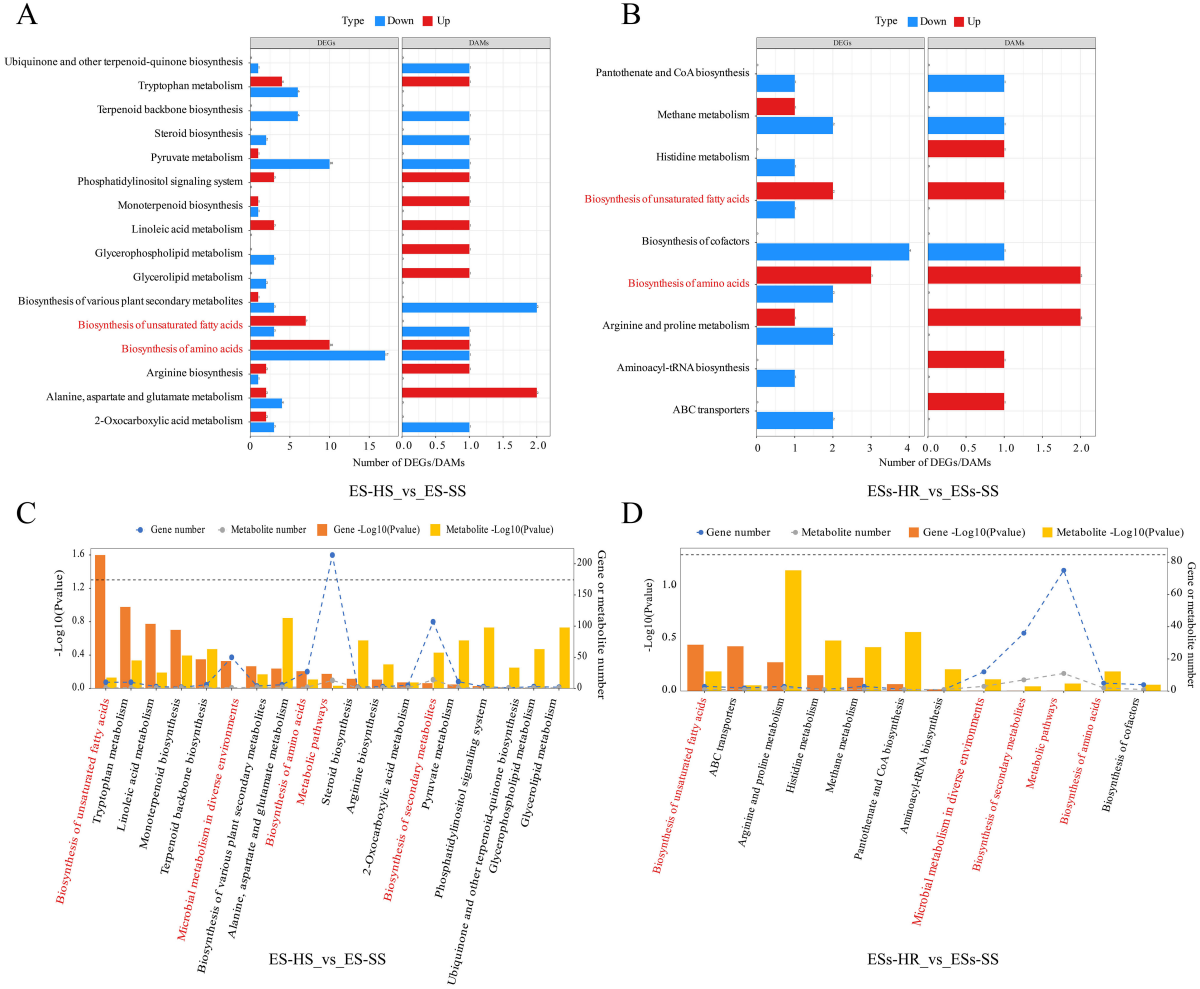
Statistical charts of KEGG pathway annotations for differentially expressed genes and differential metabolites **(A, B)**; KEGG enrichment pathway maps of differentially **(C, D)**.

**Figure 7:**
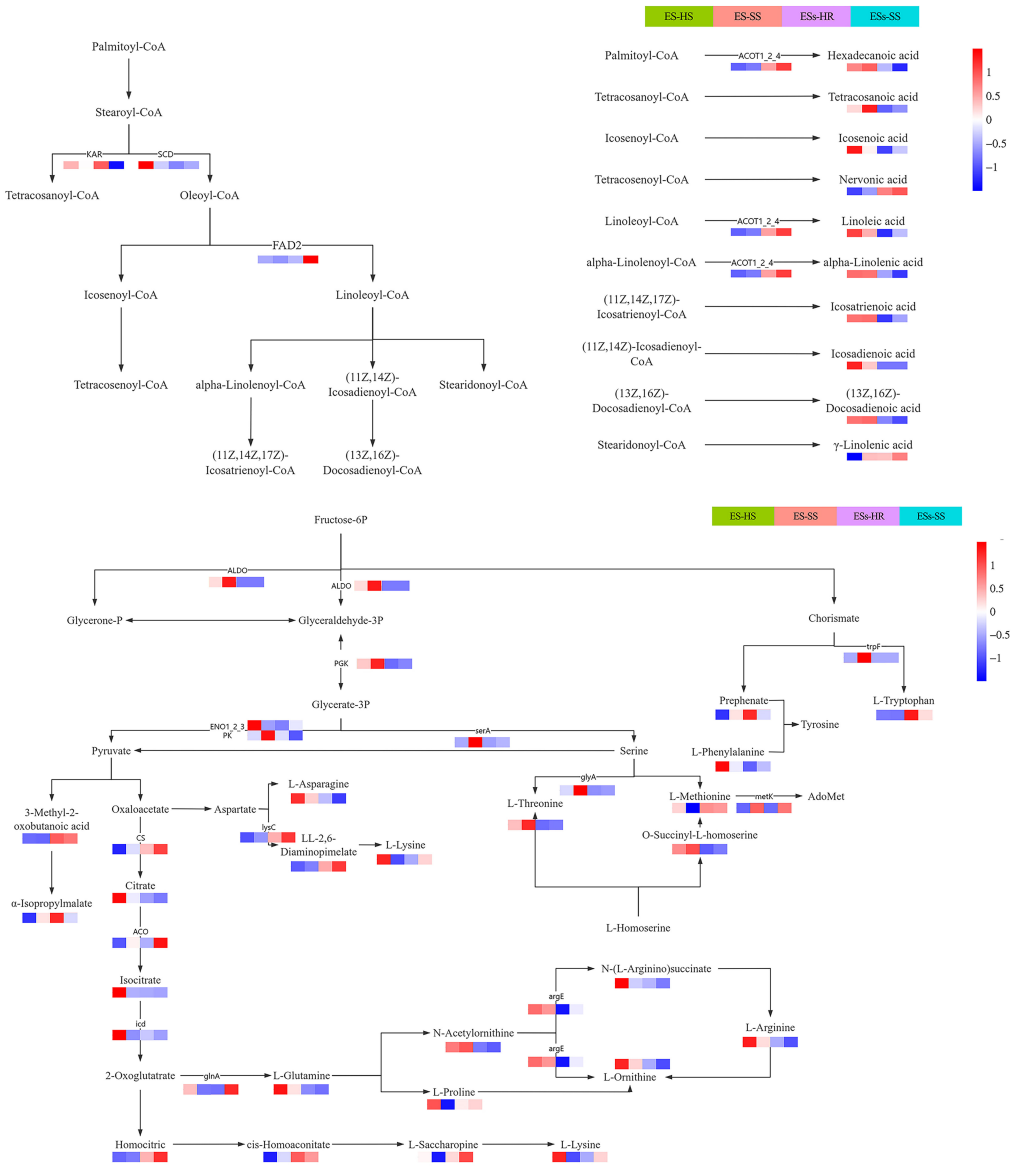
Core Pathway Mechanism Figure.

To clarify the key metabolic pathways associated with substance transport before and after grafting, DEGs and DAMs were mapped to metabolic pathways (**Figure 6**). By integrating the pathways enriched from metabolomic data with transcriptomic datasets, five pathways were identified as co-enriched, namely Biosynthesis of unsaturated fatty acids, Biosynthesis of amino acids, Microbial metabolism in diverse environments, Metabolic pathways, and Biosynthesis of secondary metabolites. The unsaturated fatty acid biosynthesis pathway is closely related to cell membrane stability and antioxidant capacity, which was highly consistent with the variation trends of antioxidant indices observed in grafted plants in this study. The amino acid metabolic pathway serves as a crucial precursor pool for the biosynthesis of pharmacologically active components. Alterations in the activity of this pathway directly affect the material basis for the synthesis of downstream target products, thereby regulating the accumulation levels of flavonoids and other related compounds. Considering the biological significance of the pathways and their relevance to the research objectives, subsequent studies will focus on these two pathways to investigate their regulatory mechanisms in depth. In the Biosynthesis of Unsaturated Fatty Acids pathway (**Figure 7A**), 10 DEGs (encodes 4 enzymes) and 10 DAMs were enriched, while in the Biosynthesis of Amino Acids pathway (**Figure 7B**), 33 DEGs (encodes 14 enzymes) and 22 DAMs were enriched (**Supplementary Tables 9**, **S10**).

## 4 Discussion

### 4.1 Directional optimization of physiological and biochemical indicators of *E.senticosus* by grafting

MC, TCA, EC and SYC are the basic physical and chemical indicators of *E.senticosus* and the quality control indicators stipulated in the Chinese Pharmacopoeia. The results showed that there was no significant difference in the four indexes between ES-SS and ES-AS, indicating that the grafting operation did not cause a general change in the basic physical and chemical indexes of ES-SS, but SYC showed rootstock specificity, and the content of this component in ES-HS was significantly down-regulated, indicating that ESs-HR did not affect the basic metabolism of ES-SS, but regulated the synthesis of syringin by interfering with specific secondary metabolic pathways, reflecting the specificity of the effect of rootstock-scion interaction on secondary metabolites. As important energy reserves of plants, SS, SP and SC are not only the basis of cell metabolism, but also osmotic adjustment substances, which are involved in the physiological process of stress resistance (Zhu et al., 2024; Xu et al., 2025). There is no significant difference in the content of the three in ES-AS and ES-SS, while the content of the three in ES-HS is significantly increased, indicating that ESs-HR can promote the accumulation of three nutrients in ES-SS and provide sufficient carbon skeleton and energy for the synthesis of secondary products, which may be related to the developed roots of *E.sessiliflorus* and the absorption of more soil nutrients (Rho et al., 2020). The stability of reactive oxygen species (ROS) is the key to normal cell metabolism and material accumulation (Nong et al., 2023). The antioxidant enzyme system composed of SOD, POD and CAT can remove excessive ROS. MDA is the end product of membrane lipid peroxidation, and its decrease indicates that the cell membrane damage is reduced. The enzyme activity in ES-HS was significantly enhanced and the MDA content was significantly reduced, indicating that ESs-HR can enhance the antioxidant capacity of ES-SS, maintain cell membrane integrity, and provide a stable environment for substance synthesis (Saud et al., 2022; Deng et al., 2025). This is consistent with the conclusion of tea tree grafting related research (Li et al., 2026). Total flavonoids, total glycosides, total polyphenols and total amino acids are important secondary metabolites and pharmacological activity basis of *E.senticosus* (Kos et al., 2025). The contents of total flavonoids, total polyphenols and total amino acids in ES-HS were significantly higher than those in ES-AS and ES-SS, indicating that ESs-HR can selectively promote the accumulation of such secondary metabolites, which may be related to the increase of nutrient accumulation (Ibrahim et al., 2010; Min et al., 2026). There was no difference in the content of total glycosides in each treatment group, suggesting that its accumulation was independently regulated or competed with other metabolic pathways.

### 4.2 The core molecular mechanism by which grafting mediates stem changes in *E.senticosus*

Transcriptome analysis showed that the effect of grafting on ES-HS transcription was much greater than that of ESs-HR. Four core pathways and 71 related genes were screened by KEGG and WGCNA, encoding 34 key proteins/enzymes. In the MAPK signaling pathway-plant ERF1 is down-regulated in ES-HS, and PP2C and SNRK2 are reversely expressed, synergistically maintaining plant homeostasis and stress resistance (Lin et al., 2021; Zhang et al., 2022; Ghanizadeh et al., 2025). CTR1, CAT and CALM in ESs-HR regulate sugar transport, oxidative homeostasis and calcium signaling. In the Peroxisome pathway, SOD and CAT were up-regulated to scavenge ROS, ACAA1 and DECR2 were down-regulated to balance metabolism and enhance antioxidant capacity, which was consistent with the previous results of antioxidant enzyme activity enhancement (Kao et al., 2017; Huang et al., 2022). In the Glycolysis/Gluconeogenesis pathway, ALDH was down-regulated in ES-HS and FBA was up-regulated in ESs-HR, which reflected that optimizing metabolic distribution provided support for ES-HS (Savchenko and Tikhonov, 2021). In the Phenylpropanoid biosynthesis pathway, the down-regulation of CYP84A in ES-HS promoted the synthesis of flavonoids (related to the increase of total flavonoids and polyphenols), and the up-regulation of POD in rootstocks enhanced the antioxidant activity and guaranteed the synthesis of substances (Shigeto and Tsutsumi, 2016). Metabolomics analysis found that in the two groups of DAMs in this study, lipids and lipid molecules accounted for the highest proportion and the most significant changes, followed by organic acids, and it was speculated that the two were the core metabolites affected by grafting. Lipids, as important macromolecules in plants, constitute the plasma membrane and participate in complex biological processes (Maryam et al., 2025). Lipids between rootstocks and scions are bidirectionally regulated: partial up-regulation can maintain cell membrane stability, optimize fluidity and enhance stress resistance (Yao, 2018); partial down-regulation reflects lipid decomposition and carbon skeleton redistribution, providing carbon source for secondary metabolite synthesis (Cha et al., 2022; Xuan et al., 2025). Organic acids and their derivatives also showed two-way changes, some of which were up-regulated to promote plant growth and material transport (Li et al., 2025), and some of the down-regulation was preferentially used for secondary metabolite synthesis (Wei et al., 2025). The KEGG pathway enrichment analysis of the two groups of DAMs showed that 12 core pathways were significantly enriched between rootstocks and scions, involving amino acid, lipid metabolism and terpenoid synthesis, indicating that grafting co-activated the regulation pathway of material accumulation in rootstocks and scions.

A comprehensive analysis of metabolomic and transcriptomic data, combined with physiological and biochemical test results, indicates that grafting significantly modulates the biosynthesis pathways of unsaturated fatty acids and amino acids between the rootstock and scion. Unsaturated fatty acids are essential components of plant cell membranes and key precursors of signaling molecules; they play a crucial role in plant growth and development, stress resistance, and metabolic regulation (He and Ding, 2020). This study identified a total of 10 DEGs in this pathway, primarily encoding four enzymes SCD, FAD2, ACOT1_2_4, and KAR, that regulate the accumulation of 10 DAMs. SCD and FAD2 are key enzyme genes; when upregulated, they catalyze the conversion of stearic acid to oleic acid and the conversion of oleic acid to linoleic acid, respectively (Zhang et al., 2019; Xue et al., 2023). This is consistent with the observed increase in linoleic acid content in ES-HS, and the elevated levels of linoleic acid promote the synthesis of downstream compounds such as alpha-linolenic acid; this synergistic effect has been verified (Paton and Ntambi, 2009; Xuan et al., 2022). Alpha-linolenic acid can generate JA via the LOX pathway, thereby activating the expression of defense genes and inducing the accumulation of secondary metabolites (Kimberlin et al., 2022), a finding consistent with the results of physiological and biochemical experiments. A decrease in ACOT1_2_4 leads to a reduction in hexadecanoic acid levels, thereby avoiding metabolic competition and energy waste (Chen et al., 2021). While KAR levels increased, downstream hexadecanoic acid decreased in ES-HS but increased in ESs-HR, suggesting that it may be transported to ESs-HR to enhance drought resistance. Consistent with the research group’s previous findings (Wang, 2023), unsaturated fatty acid levels tended to decrease in ES-HS and increase in ESs-HR; it is hypothesized that these changes serve to enhance the rootstock’s resistance and support scion growth, respectively. The key role of amino acids lies in their function as precursors for protein synthesis and as donors of carbon and nitrogen skeletons for secondary metabolites such as flavonoids (Yokoyama, 2024). In this study, 33 DEGs were enriched in the “Biosynthesis of amino acids” pathway; these genes encode 14 related enzymes that regulate the accumulation of 22 DAMs. Enzymes associated with glycolysis, such as ALDO and PGK, were significantly downregulated in ES-HS, suggesting that grafting weakens its energy metabolism and carbon skeleton breakdown. Intermediate metabolites (such as increased levels of citrate and isocitrate) are redirected toward the synthesis of amino acids and secondary metabolites (Timm and Arrivault, 2021), consistent with the increased levels of six functional amino acids (L-arginine, L-asparagine, L-ornithine, L-glutamine, L-methionine, and L-tryptophan) in ES-HS. The accumulation of these amino acids contributes to enhancing the stress resistance of ES-HS (Shi et al., 2013; Cohen et al., 2017) and promoting protein synthesis (Lee et al., 2023), among other effects; their accumulation is one of the reasons for the increase in nutrient content. Downregulation of glnA inhibits L-threonine synthesis, indirectly promoting the accumulation of L-lysine and other amino acids; synergistic regulation by glnA, trpF, and other genes results in increased L-saccharopine, L-phenylalanine, and L-proline levels in the ES-HS, and L-lysine in ES-HS, while decreasing them in ESs-HR. In ESs-HR, more amino acids are consumed for root growth, enhanced lignification, and maintenance of the plant’s structural integrity; alternatively, ESs-HR may transport more amino acids to ES-HS to enhance its growth and development, improve stress resistance, and promote the synthesis of secondary metabolites (Saud et al., 2022), which directly correlates with the increased total polyphenol and total flavonoid contents in the ES-HS. This finding is consistent with the research group’s previous studies, suggesting that the grafting system regulates nitrogen metabolic balance (Wang et al., 2023).

## 5 Conclusions

This study examined grafted plants using *E.senticosus* as the scion and *E.sessiliflorus* as the rootstock, and found that grafting significantly enhanced the antioxidant capacity, nutrient content, and secondary metabolite levels in the stems of *E.senticosus*. In-depth analysis revealed that the most significant changes in lipids and organic acids occurred in the ES-HS_*vs*_ES-SS and ESs-HR_*vs*_ESs-SS comparisons, with both sets of changes concentrated in 12 pathways, including amino acid metabolism and lipid metabolism. Transcriptomic analysis identified four core pathways: MAPK signaling pathway-plant, Glycolysis/Gluconeogenesis, Phenylpropanoid biosynthesis, Peroxisome pathway, involving 71 key genes (including *TRINITY_DN27179_c0_g1*) that encode 34 proteins/enzymes such as PR1. Further integrated analysis identified two key pathways; the expression of 10 key enzyme genes in the unsaturated fatty acid biosynthesis pathway plays a regulatory role in the accumulation of Linoleic acid, alpha-Linolenic acid, Icosenoic acid, Icosadienoic acidin ES-HS. The expression of 33 key enzyme genes in amino acid biosynthetic pathways contributes to the regulation of L-arginine, L-glutamine, L-tryptophan, L-asparagine, L-ornithine, L-methionine, L-proline, L-lysine, L-phenylalanine, and L-saccharopine accumulation in ES-HS. These two pathways provide preliminary insights into the mechanisms by which grafting influences changes in the composition of *E.senticosus* stems.

## Data Availability

Transcriptomics: The raw sequence data reported in this paper have been deposited in the Genome Sequence Archive (Genomics, Proteomics & Bioinformatics 2025) in National Genomics Data Center (Nucleic Acids Res 2026), China National Center for Bioinformation / Beijing Institute of Genomics, Chinese Academy of Sciences (GSA: CRA046202) that are publicly accessible at https://ngdc.cncb.ac.cn/gsa. Metabolomics: The data reported in this paper have been deposited in the OMIX, China National Center for Bioinformation / Beijing Institute of Genomics, Chinese Academy of Sciences (https://ngdc.cncb.ac.cn/omix: accession no.OMIX018440, OMIX018441, OMIX018442, OMIX018443).

## Supplementary material

Supplementary Figure 1 Grafting diagram.

Supplementary Figure 2 Enrichment Analysis Plot for the 7 Major Modules in WGCNA.

Supplementary Figure 3 Expression of DEGs in the Secondary Metabolite Biosynthesis Pathway of Samples. **(A)**: qRT-PCR validation results; **(B)**: RNA-seq sequencing results.

Supplementary Figure 4 Total ion chromatograms. **(A–D)** are the total ion chromatograms of the four sample groups in positive ion mode; **(E–H)** are the total ion chromatograms of the four sample groups in negative ion mode. In each panel, each sample is displayed in a different manner.

Supplementary Figure 5 OPLS-DA model and 200 permutation verification diagram.

Supplementary Table 1 Primers for qRT-PCR analysis.

Supplementary Table 2 Transcriptome Data Quality Control Statistics.

Supplementary Table 3 Mapping ratio statistics.

Supplementary Table 4 The top 30 genes by number of intra-module connections.

Supplementary Table 5 Related genes and functional annotation.

Supplementary Table 6 Summary of Differentially Accumulated Metabolites Identified in ES-HS_*vs*_ES-SS.

Supplementary Table 7 Summary of Differentially Accumulated Metabolites Identified in ES-HS_*vs*_ES-SS.

Supplementary Table 8 Expression Pattern Distribution of DAMs in ES-HS_*vs*_ES-SS and ESs-HR_*vs*_ESs-SS Comparison Groups.

Supplementary Table 9 Expression Patterns of DEGs and DAMs in the Biosynthesis of Unsaturated Fatty Acids Pathway.

Supplementary Table 10 Expression Patterns of DEGs and DAMs in the Biosynthesis of amino acids Pathway.
